# Orthogonal canalized polaritons via coupling graphene plasmon and phonon polaritons of hBN metasurface

**DOI:** 10.1515/nanoph-2025-0385

**Published:** 2025-11-03

**Authors:** Chia-Chien Huang

**Affiliations:** 34916 Department of Physics and Graduate Institute of Nanoscience, National Chung Hsing University, 145 Xingda Rd., South Dist., Taichung City 40227, Taiwan

**Keywords:** canalization, phonon polaritons, plasmon polaritons, hyperbolic dispersions

## Abstract

Metasurfaces composed of van der Waals materials exhibit extreme anisotropy and strong subwavelength confinement, enabling precise control of mid-infrared and terahertz waves for advanced photonic and optoelectronic applications. Among their intriguing phenomena, canalization – characterized by nearly diffraction-free propagation – offers significant potential for nanoscale light manipulation and enhanced light–matter interactions. Recently, gratings were demonstrated to induce synthetic transverse optical (STO) resonances, facilitating canalization perpendicular to the ribbon axis. In this study, we introduce a novel canalization mechanism by sandwiching a grating of hBN ribbons between graphene layers. The hybrid structure achieves orthogonal redirection of STO-induced canalization through the coupling plasmon polaritons in graphene and phonon polaritons in the hBN ribbons, achieving beam widths of approximately 300 nm (∼*λ*_0_/20, where *λ*_0_ is the free-space wavelength) across the spectral range of 1,470–1,510 cm^−1^. Detailed analyses were conducted by varying graphene’s Fermi energy and geometric parameters, elucidating key field characteristics and spatial evolution of the canalization. Moreover, practical feasibility is demonstrated through simulated experimental antenna-launched excitation. Our finding holds promise for the development of polariton canalizations in diverse vdW material systems and facilitating on-chip photonic applications.

## 1 Introduction

Nanophotonics has rapidly progressed over the past few decades, driven primarily by advances in material science, fabrication techniques, and deeper insights into nanoscale light–matter interactions [1], [2], [3], [4]. Among the variety of materials explored, two-dimensional (2D) van der Waals (vdW) materials have emerged as outstanding platforms due to their unique abilities to strongly couple photons with intrinsic excitations, including plasmons, phonons, and excitons [5], [6], [7], [8], [9]. For instance, surface plasmon polaritons (SPPs) [10], [11], [12], [13], formed through photon coupling to collective electronic oscillations at metal-dielectric interfaces, can tightly confine electromagnetic fields and enhance local optical intensities significantly. Similarly, phonon polaritons (PhPs) [14], [15], [16], [17], [18], [19], [20], [21], [22], [23], [24], [25], [26], [27], [28], [29], arising from photon coupling to lattice vibrations in polar crystals, have been shown to exhibit exceptionally low damping compared to SPPs, offering advantages for applications requiring sustained propagation and high field confinement in the mid-infrared (mid-IR) and terahertz regimes. PhPs in vdW polar crystals, such as hexagonal boron nitride (hBN) [14], [15], [16], [17], [18] and α-phase molybdenum trioxide (α-MoO_3_) [19], [20], [21], [22], [23], [24], [25], [26], [27], [28], [29], demonstrate remarkable optical anisotropy due to hyperbolic dispersion. This hyperbolic behavior, characterized by opposite signs of the permittivity along orthogonal crystal axes, gives rise to exotic optical phenomena including negative refraction [30], [31], [32], [33], [34], super-resolution imaging [35], [36], [37], and notably canalization [19], [20], [21], [22], [23], [24], [25], [26], [27], [28], [29], [38], [39], [40], [41], [42]. Canalization, originating from a topological transition of isofrequency contours (IFCs) between hyperbolic and elliptical dispersion regimes, allows nearly collimation propagation of electromagnetic energy along highly collimated directions, making it highly attractive for applications such as nanoscale optical circuits, molecular sensing, and high-resolution imaging.

Polariton canalization has been explored extensively in α-MoO_3_-based heterostructures, including twisted bilayers [19], [20], [21], [22] or trilayers [25], graphene-covered α-MoO_3_ slabs [23], [24], [41], α-MoO3 deposited on 4H-silicon carbide (SiC) substrates [28], [29], and LiV_2_O_5_ slab placed on silicon dioxide (SiO_2_) substrate [42]. Prior canalization platforms offer complementary strengths. Twisted α-MoO_3_ bilayers [19], [20], [21], [22], [23] achieve canalization within frequency-dependent angle windows, reducing the need for an exact twist while still requiring controlled twist engineering. Graphene-α-MoO_3_ has demonstrated well-collimated canalization [24], though it lacks the same breadth of tunable structural parameters. α-MoO_3_/4H-SiC canalization has been experimentally realized [28], underscoring the robustness of phonon-polaritonic canalization enabled by substrate coupling, even in the presence of high-order modes. In an alternative design, metasurfaces composed of van der Waals (vdW) materials [38], [39], [43] provide additional degrees of freedom to tailor polariton dispersion and spatial confinement, albeit with greater fabrication complexity. Li et al. [39] recently reported a remarkable canalization phenomenon in a grating structure composed of hBN ribbons, attributed to synthetic transverse optical (STO) resonances. Below the STO resonance [43], the hBN ribbons exhibit an in-plane hyperbolic dispersion due to weak near-field polariton coupling along the ribbons. In contrast, when the working frequency exceeds the STO resonance [39], strong near-field coupling induces elliptical dispersion, resulting in canalized propagation across the ribbons. In addition, other relevant studies focusing on the polariton canalizations include the moiré hyperbolic plasmons in pairs of hyperbolic metasurfaces by rotating two coupled metasurfaces [44], ghost phonon polaritons being both propagating and evanescent for a long-distance [45], and hyperbolic surface PhPs with temperature-controlled dispersion engineering in a non-hyperbolic crystal [46].

In this work, we propose and theoretically investigate a novel approach to achieving high-quality, actively tunable polariton canalization by sandwiching an hBN metasurfaces between graphene layers. This proposed heterostructure significantly modifies the optical response of the bare hBN metasurface, giving rise to strong hybrid plasmon–phonon polariton (HPPhP) coupling [47], [48]. Such coupling drastically alters the dispersion relations and electric field distributions, enabling a previously unexplored canalization phenomenon that propagates orthogonally to the direction reported in traditional STO-induced systems. In contrast to earlier canalization platforms, the graphene-hBN-graphene metasurface integrates electrostatic tunability and lithographically defined anisotropy, thereby enabling reconfigurable beam steering – specifically, orthogonal redirection – without the need for twist engineering. By systematically examining the dependence of canalization characteristics on geometric parameters, operating frequencies, and graphene Fermi energies, we provide comprehensive insight into the physical mechanisms governing the proposed canalization mode. Furthermore, we evaluate the spatial evolution and field confinement of the canalized beams, demonstrating their high quality and subwavelength-scale confinement capabilities. Ultimately, this study not only introduces a novel method for realizing highly collimated, broadband, and actively tunable polariton canalization but also lays the foundation for exploiting hybrid vdW heterostructures in advanced photonic applications.

## 2 Polariton canalization in graphene-covered hBN metasurfaces

### 2.1 hBN metasurfaces without graphene layers

**Figure 1:**
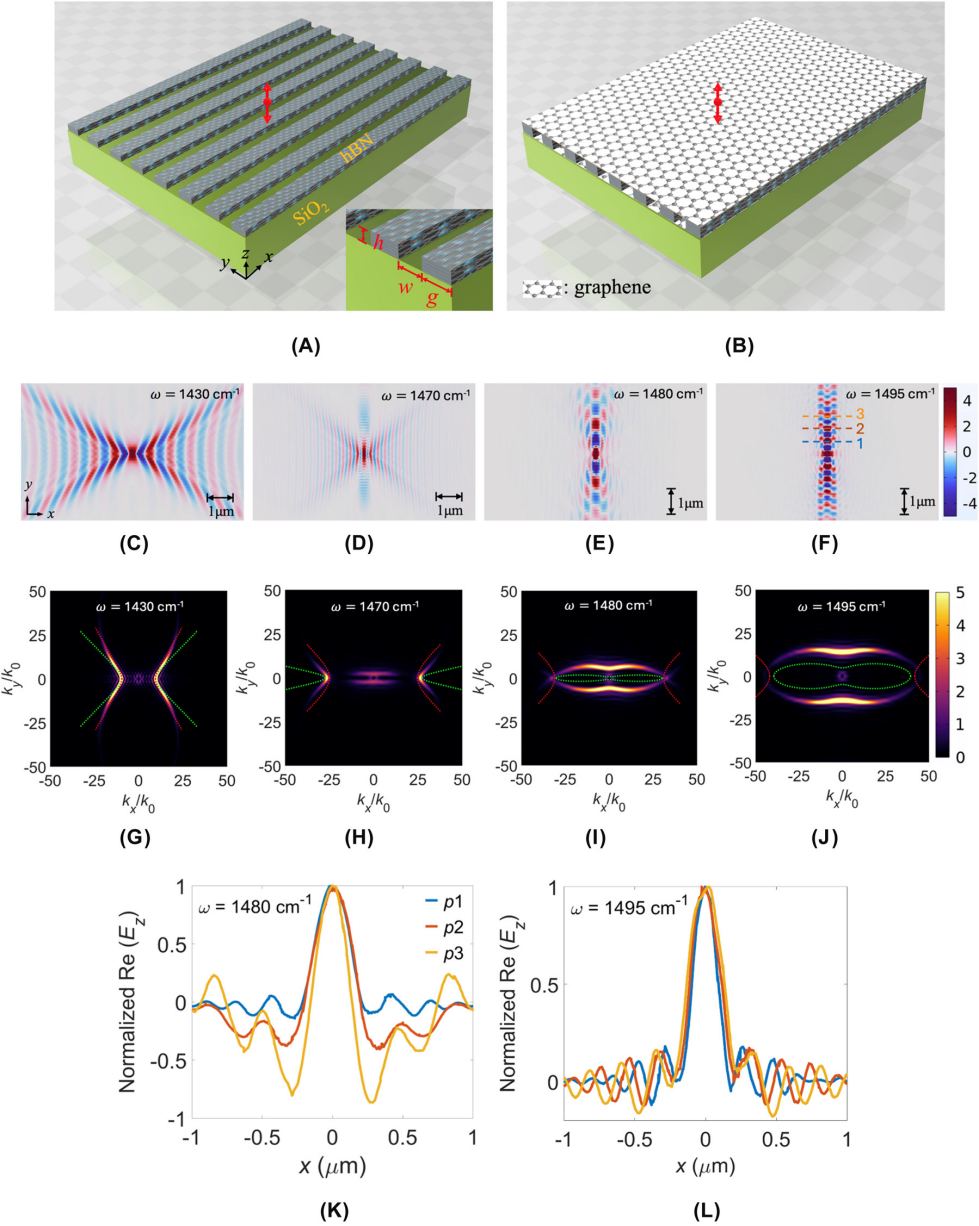
Schematic illustrations of bare and graphene-sandwiched hBN metasurfaces, and the polariton behavior of the bare hBN metasurface. (A) Bare hBN metasurface on a SiO_2_ substrate. (B) Graphene-sandwiched hBN metasurface on a SiO_2_ substrate. Geometric parameters are defined as the grating period (*P*), ribbon gap (*g*), ribbon width (*w*), and ribbon height (*h*). A double-arrow symbol indicates the location and polarization of the dipole source. Simulated electric-field distributions, Re(*E*_*z*_), for the bare hBN metasurfaces at frequencies *ω*: (C) 1,430, (D) 1,470, (E) 1,480, and (F) 1,495 cm^−1^, with corresponding IFCs shown in (G)–(J). Simulations are conducted for parameters *P* = 100 nm, *w* = 70 nm, *g* = 30 nm, and *h* = 20 nm. The analytical IFCs calculated by MEMT and MGA approaches are indicated by green and red dotted lines, respectively. Re(*E*_*z*_) profiles along the *x*-direction at positions 1, 2, and 3 indicated in (F), for *ω* = (K) 1,480 and (L) 1,495 cm^−1^.

The frequency-dependent permittivity of an hBN slab can be described by the Lorentz model (see Eq. (S1) in Note 1 of Supplementary Material) [39], [43]. Within Reststrahlen band II (*ω* = 1,395–1,630 cm^−1^), hBN flake naturally supports strongly confined volume PhPs. Prior to investigating graphene-sandwiched hBN metasurfaces, we re-examine the canalization mechanism of bare hBN metasurface with uniaxial anisotropy placed on a SiO_2_ substrate, as illustrated in Figure 1(A). The geometrical parameters defining this structure are the grating period (*P*), gap between adjacent ribbons (*g*), ribbon width (*w*), height (*h*), and the duty cycle (*η* = *w*/*P*). Each hBN ribbon is oriented along the *x*-direction. An electric dipole source, polarized along the *z*-direction, is positioned 50 nm above the metasurface, indicated by the double-arrow symbol. After patterning, the hBN metasurface exhibits biaxially anisotropic permittivity, described by an effective permittivity tensor $\tilde{\varepsilon }$ = diag{*ε*_eq,*x*_, *ε*_eq,*y*_, *ε*_eq,*z*_}. To accurately account for the strong polaritonic near-field coupling among ribbons, the modified effective medium theory (MEMT) [38], [39], which incorporates a nonlocal correction *ε*_*c*_, is employed. According to MEMT, the equivalent permittivity components are given by:

(1) $$\varepsilon_{eq,x}=\eta \varepsilon_{x}+\left(1-\eta \right)\varepsilon_{a},$$

(2) $$\varepsilon_{eq,y}={\left(\frac{\eta }{\varepsilon_{y}}+\frac{1-\eta }{\varepsilon_{c}}\right)}^{-1},$$

(3) $$\varepsilon_{eq,z}=\eta \varepsilon_{z}+\left(1-\eta \right)\varepsilon_{a},$$

where *ε*_*x*_, *ε*_*y*_, and *ε*_*x*_ intrinsic permittivity components along the *x*, *y*, and *z* directions, respectively, *ε*_*a*_ = 1 denotes the permittivity of air, and the nonlocal correction term *ε*_*c*_ = (2*P*/*πh*) ln [csc(*πg*/2*P*)] [38], [39] accounts for the strong near-field coupling, treating the metasurface as a spatially dispersive medium. In contrast, the Maxwell–Garnett approximation (MGA) [43], [49], which only considers weak near-field coupling, replaces *ε*_*c*_ with *ε*_*a*_. The calculated permittivity components using MEMT and MGA are presented in Figure S1 of Supplementary Material.

To validate the presence of the STO resonance and explore the canalization phenomenon numerically, an electric dipole source with polarization along the *z*-direction is located at 50 nm above the hBN metasurface. We calculated the *z*-component electric field Re(*E*_*z*_) at 20 nm above the hBN metasurface [24], [32], [34], [41], for frequencies *ω* = 1,430, 1,470, 1,480, and 1,495 cm^−1^ (Figure 1(C)–(F), respectively), using parameters *P* = 100 nm, *w* = 70 nm, *g* = 30 nm (*η* = 0.7), and *h* = 20 nm. Corresponding isofrequency contours (IFCs) in wavevector space (*k*_*x*_, *k*_*y*_) (Figure 1(G)–(J)) were computed by performing Fourier transforms of the numerically obtained Re(*E*_*z*_) fields with spatial resolutions *N*_*x*_ = 500 and *N*_*y*_ = 500 in COMSOL Multiphysics [32], [41]. Analytical IFC solutions based on a three-layer slab waveguide model [50] [see Supplementary Material, Eq. (S10)] using MEMT (green dotted lines) and MGA (red dotted lines) permittivities are included for comparison. At *ω* = 1,430 cm^−1^, numerical results clearly show a bare hyperbolic dispersion profile (Figure 1(C)), and the analytical IFC with MGA closely matches numerical data (Figure 1(G)). As frequency increases to *ω* = 1,470 cm^−1^, strong near-field coupling emerges, marking the onset of the STO resonance (Figure 1(D) and (H)). At *ω* = 1,480 cm^−1^, canalization dominated by the STO resonance is clearly observed (Figure 1(E) and (I)), with enhanced confinement at *ω* = 1,495 cm^−1^ (Figure 1(F) and (J)).

Note that analytical IFCs with MGA gradually deviate from numerical results as frequency increases, indicating its failure at strong near-field coupling regimes. By contrast, MEMT provides improved predictions at higher frequencies (*ω* ≥ 1,480 cm^−1^), correctly capturing the elliptical dispersion topology induced by STO resonance (Figure 1(I) and (J)), though with noticeable inaccuracies at lower frequencies. The results show that analytical IFCs based on a three-layer slab waveguide model [50] with MGA or MEMT are failed to capture accurate hyperbolic and elliptical IFCs for the present hBN grating configuration. We know that the slab model can obtain exact solutions for a slab waveguide structure. As a result, we calculate the numerical and analytical IFCs adopting the same permittivities calculated from MEMT [Eqs. (1)–(3)], to examine the accuracy of the numerical solutions. The numerically obtained Re(*E*_*z*_) fields at frequencies *ω* = 1,430, 1,465, 1,470, 1,475, 1,480, and 1,495 cm^−1^, along with their IFCs, are presented in Figure S3(a)–(l) of Supplementary Material, respectively. The results show excellent agreement for the IFCs obtained from the numerical and analytical solutions across all tested frequencies. To evaluate the energy dissipation, the one-dimensional (1D) profiles of Re(*E*_*z*_) and the corresponding decay rates along the *y*-direction at *ω* = 1,475, 1,480, and 1,495 cm^−1^ are presented in Figure S4(a). Additionally, we quantitatively evaluated the canalized fields by analyzing 1D Re(*E*_*z*_) distributions along the dashed lines indicated in Figure 1(F), shown in Figure 1(K) and (L) for *ω* = 1,480 and 1,495 cm^−1^, respectively. The field distributions exhibit significant side-lobe ripples, resulting in high crosstalk with adjacent channels. Notably, at position 3, the amplitude of side-lobed ripples even surpasses the central lobe for *ω* = 1,480 cm^−1^. At *ω* = 1,495 cm^−1^, the side-lobe amplitude remains approximately 20 % of the central peak amplitude. The analysis demonstrates substantial room for improvement by the proposed structure, significantly reducing side-lobe ripples and crosstalk.

### 2.2 Proposed graphene-sandwiched hBN metasurface

**Figure 2:**
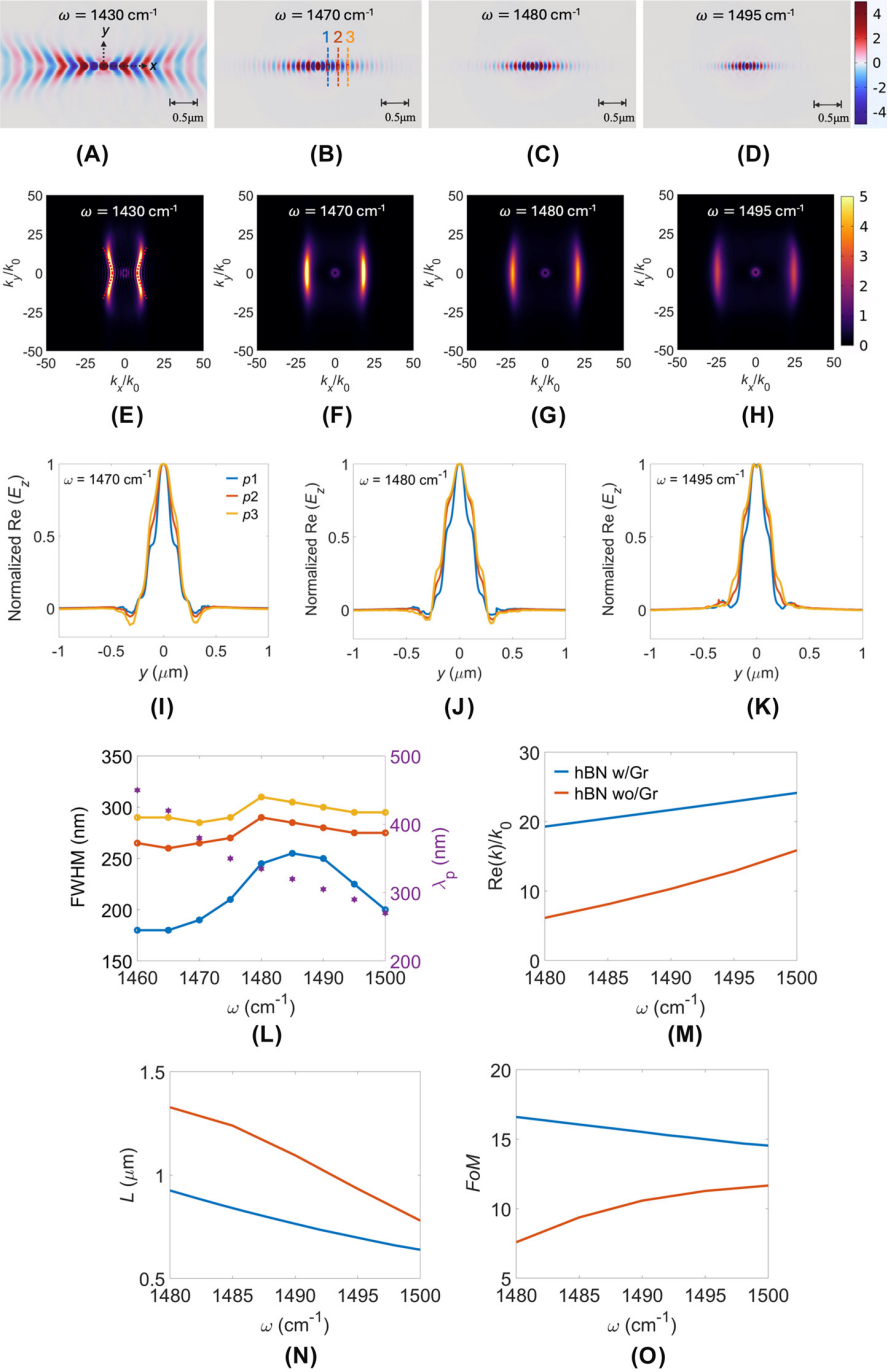
Polariton canalization in graphene-sandwiched hBN metasurface. Simulated electric-field distributions Re(*E*_*z*_) at frequencies *ω*: (A) 1,430, (B) 1,470, (C) 1,480, and (D) 1,495 cm^−1^, along with their corresponding IFCs shown in (E)–(H). (I)–(K) Detailed Re(*E*_*z*_) profiles at three positions (*p*1 to *p*3) indicated in (B) for *ω* = 1,470, 1,480, and 1,495 cm^−1^, respectively. (L) Full width at half maximum (FWHM) and polariton wavelength (*λ*_*p*_) versus frequency, assessing canalization quality. Mode characterization: (M) real part of effective refractive index Re(*k*)/*k*_0_, (N) propagation length *L* = 1/Im(*k*), and (O) figure of merit *FoM* = Re(*k*)/Im(*k*) versus operating frequency for the hBN metasurface with (w/) and without (wo/) graphene (Gr). Simulation parameters are *P* = 100 nm, *w* = 70 nm, *g* = 30 nm, *h* = 20 nm, and *E*_*f*_ = 0.19 eV.

To achieve novel and tunable polariton canalization, we propose sandwiching the hBN metasurface between graphene layers, as illustrated in Figure 1(B). Graphene’s optical properties, governed by its surface conductivity derived from the Kubo formula [19], [21], [22], [44], [45], [46] [Supplementary Material, Eq. (S2)], can be actively tuned by adjusting its Fermi energy (*E*_*f*_) via electrostatic gating or chemical doping. In our simulations, we place an electric dipole source polarized along the *z*-direction at 50 nm above the upper graphene layer. Using the parameters *P* = 100 nm, *w* = 70 nm, *g* = 30 nm, *h* = 20 nm, and *E*_*f*_ = 0.19 eV, we calculate the spatial field distributions of Re(*E*_*z*_) at a probing height of 20 nm above the upper graphene layer for frequencies *ω* = 1,430, 1,470, 1,480, and 1,495 cm^−1^, shown in Figure 2(A)–(D), respectively, alongside their corresponding IFCs presented in Figure 2(E)–(H). We observe that the hyperbolic field profile of the proposed graphene-sandwiched hBN metasurface exhibits a significantly smaller opening angle (Figure 2(A)) compared to the bare hBN metasurface (Figure 1(C)) at *ω* = 1,430 cm^−1^, where the opening angle *ψ* is the angle [tan(*ψ*/2) = √(−*ε*_*y*_/*ε*_*x*_)] [51] between the two polaritonic beams of the V-shaped hyperbolic field profile, which is formed by the real-space energy flow. This arises from the coupling between the isotropic dispersion of graphene and the hyperbolic dispersion of the hBN metasurface, which modifies the IFC from a hyperbola with a larger opening angle to one with a smaller angle (see Figures 1(G) and 2(E)).

As the STO resonance near *ω* = 1,470 cm^−1^ in the bare hBN metasurface, it generates polariton canalization in the *y*-direction through near-field coupling between hBN ribbons, analogous to a nanoparticle chain mechanism. When graphene layers are added, the isotropic SPPs of graphene hybridize with the anisotropic PhPs of hBN metasurface. The graphene layers contribute an additional negative in-plane permittivity, significantly modifying the effective in-plane permittivities of the hBN metasurface. At certain *E*_*f*_, the originally dominant negative permittivity component of hBN metasurface can flip sign, resulting in the effective anisotropy ratio of hBN metasurface, changing the balance between its principal permittivity components. This adjustment in anisotropy can reorient the principal axes of the IFCs, so that the canalization orientation rotates to its orthogonal one compared with that in bare hBN metasurface. Moreover, due to the orientation of hBN ribbons in the *x*-direction, the polariton canalization along this direction is further enhanced, benefiting from a plasmonic waveguiding effect.

To characterize the energy dissipation, we plotted 1D Re(*E*_*z*_) field profiles along the propagation direction of canalization at *ω* = 1,470, 1,480, and 1,495 cm^−1^ (see Figure S4(b)). As expected, higher frequencies exhibit faster decay rates of the electric field amplitude. Additionally, we show the profiles at positions 1–3 for *ω* = 1,470, 1,480, and 1,495 cm^−1^ (Figure 2(I)–(K)), clearly illustrating that the graphene-sandwiched hBN metasurface significantly suppresses side lobes that typically occur in bare hBN metasurfaces, confirming the superior quality of canalization. To quantitively evaluate the beam width versus frequency, defined by full width at half maximum (FWHM), we show the results in Figure 2(L). At *ω* = 1,480 (1,495) cm^−1^, the FWHMs from positions 1 to 3 are 245 (225), 290 (275), and 305 (295) nm, respectively, while the polariton wavelengths (*λ*_*p*_) are 335 (290) nm, marked with hexagonal symbols. Notably, the 20 nm incremental increase in FWHM (∼20 nm from *p*1 to *p*3) remains consistently small, indicating excellent beam collimation over propagation distances. Furthermore, we present analyses of mode confinement [Re(*k*)], propagation distance [*L* = 1/Im(*k*)], and figure of merit [*FoM* = Re(*k*)/Im(*k*)] in Figure 2(M)–(O), respectively. Despite of possessing shorter *L* relative to the bare hBN metasurface (hBN wo/Gr), our proposed structure (hBN w/Gr) exhibits superior mode confinement and higher *FoM*, indicating enhanced relative propagation length (*FoM*). Our proposed design demonstrates enhancements in three key aspects: minimal side-lobe ripples, improved mode confinement, and increased *FoM*. To further verify the accuracy of the numerical simulations, we derived analytical dispersion relations using a five-layer slab waveguide model (Supplementary Material, Eq. (S9), Figure S2). We calculate the numerical and analytical IFCs adopting the same permittivities calculated from MEMT. Numerical solutions of this model are provided in Figure S5(a)–(f) (Re(*E*_*z*_) fields) and g–l (IFCs) of Supplementary Material for *ω* = 1,430–1,495 cm^−1^, demonstrating excellent agreement with the analytical predictions.

## 3 Influence of graphene Fermi energy

**Figure 3:**
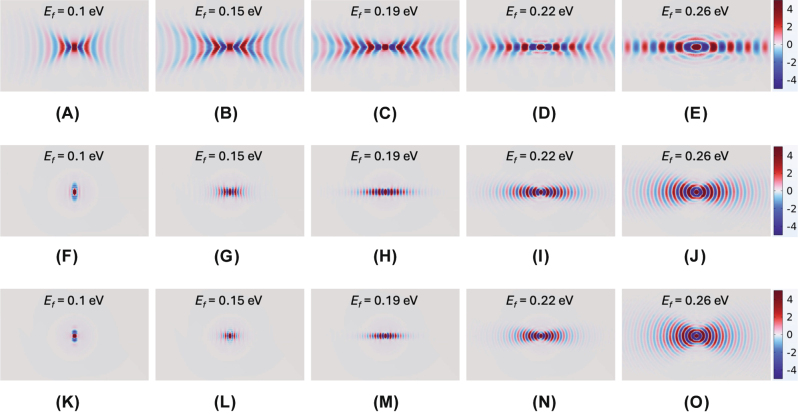
Effect of graphene Fermi energy on canalization characteristics. Simulated Re(*E*_*z*_) field distributions illustrating the influence of graphene Fermi energy at frequencies *ω* = 1,430 cm^−1^ for *E*_*f*_ values of (A) 0.10, (B) 0.15, (C) 0.19, (D) 0.22, and (E) 0.26 eV; (F)–(J) *ω* = 1,480 cm^−1^ for *E*_*f*_ values of (F) 0.10, (G) 0.15, (H) 0.19, (I) 0.22, and (J) 0.26 eV; (K)–(O) *ω* = 1,495 cm^−1^ for *E*_*f*_ values of (K) 0.10, (L) 0.15, (M) 0.19, (N) 0.22, and (O) 0.26 eV. The geometric parameters are fixed as follows: *P* = 100 nm, *w* = 70 nm, *g* = 30 nm, and *h* = 20 nm.

The HPPhP field profile of the proposed system was analyzed versus graphene’s *E*_*f*_ at frequencies *ω* = 1,430, 1,480, and 1,495 cm^−1^ (see Figure 3). At *ω* = 1,430 cm^−1^, increasing *E*_*f*_ gradually transforms the hyperbolicity to the ellipticity, reaching an optimal condition at *E*_*f*_ = 0.26 eV (see Figure 3(E)); however, even at optimal conditions, substantial diffraction and residual elliptical dispersion toward the *y*-direction persist, limiting canalization quality. In stark contrast, at frequencies near the STO resonance of *ω* = 1,480 cm^−1^, increasing *E*_*f*_ beyond 0.15 eV effectively redirects energy flow along the *x*-direction, significantly improving the collimation of canalization. Optimal canalization occurs at *E*_*f*_ = 0.19 eV (see Figure 3(H) and (M) for *ω* = 1,480 and 1,495 cm^−1^, respectively), clearly exhibiting a topological transition. Further increasing *E*_*f*_ beyond this critical value leads to dominant elliptical dispersion, causing the canalization to disappear (see Figure 3(I), (J), (N), and (O)). These findings highlight the proposed graphene-hBN metasurface structure as a highly promising platform for actively tunable polariton canalization, providing significant opportunities for advanced nanophotonic.

**Figure 4:**
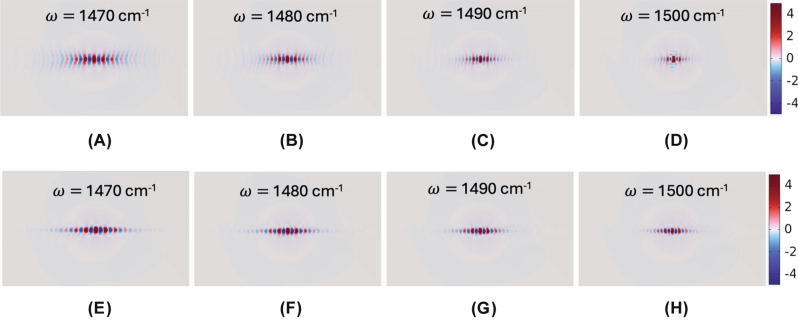
Effect of graphene Fermi energy for a larger *P*. Simulated Re(*E*_*z*_) field distributions for periodicity *P* = 150 nm (*w* = 105 nm, *g* = 45 nm, maintaining duty cycle *η* = 0.7) at frequencies *ω* = (A) 1,470, (B) 1,480, (C) 1,490, and (D) 1,500 cm^−1^ with *E*_*f*_ = 0.19 eV, and (E)–(H) corresponding improved canalization at elevated *E*_*f*_ = 0.22 eV. Ribbon height is at *h* = 20 nm.

Next, we examine the effect of varying the periodicity (*P*) while maintaining the duty cycle (*η* = 0.7). Increasing the periodicity to *P* = 150 nm (*w* = 105 nm, *g* = 45 nm), we calculated field distributions of Re(*E*_*z*_) for frequencies *ω* = 1,470, 1,480, 1,490, and 1,500 cm^−1^ (Figure 4(A)–(D), respectively) at *h* = 20 nm and *E*_*f*_ = 0.19 eV. Compared to the smaller periodicity of *P* = 100 nm, the larger-structured metasurfaces exhibit relatively moderate hyperbolic dispersion, arising from stronger near-field coupling between hBN ribbons. Consequently, canalization quality at *E*_*f*_ = 0.19 eV is somewhat detrimental for the larger *P*. However, increasing the graphene Fermi level to *E*_*f*_ = 0.22 eV enhances the coupling strength between graphene PPs and ribbon-confined PhPs, significantly improving canalization quality, as demonstrated in Figure 4(E)–(H). Experimentally, larger ribbon dimensions relax fabrication precision requirements, thereby offering practical advantages for device realization. Moreover, we also present the Re(*E*_*z*_)’s for varying hBN ribbon thicknesses in Figure S6(a)–(d), with the corresponding IFCs in Figure S6(e)–(h) at parameters: *ω* = 1,480 cm^−1^, *P* = 100 nm, *w* = 70 nm, *g* = 30 nm, and *E*_*f*_ = 0.19 eV. The results reveal a clear topological transition at *t* = 20 nm. Specifically, for thinner ribbons (*t* = 15 nm, Figure S6(a) and (e)), elliptical dispersion dominates due to stronger coupling between graphene SPPs at the top and bottom surfaces. Conversely, thicker ribbons (*t* = 30 and 50 nm, Figure S6(b), (d), (f), and (h)) exhibit predominantly hyperbolic dispersion, indicating weaker coupling.

## 4 Simulated antenna-launched canalization in realistic experimental setups

**Figure 5:**
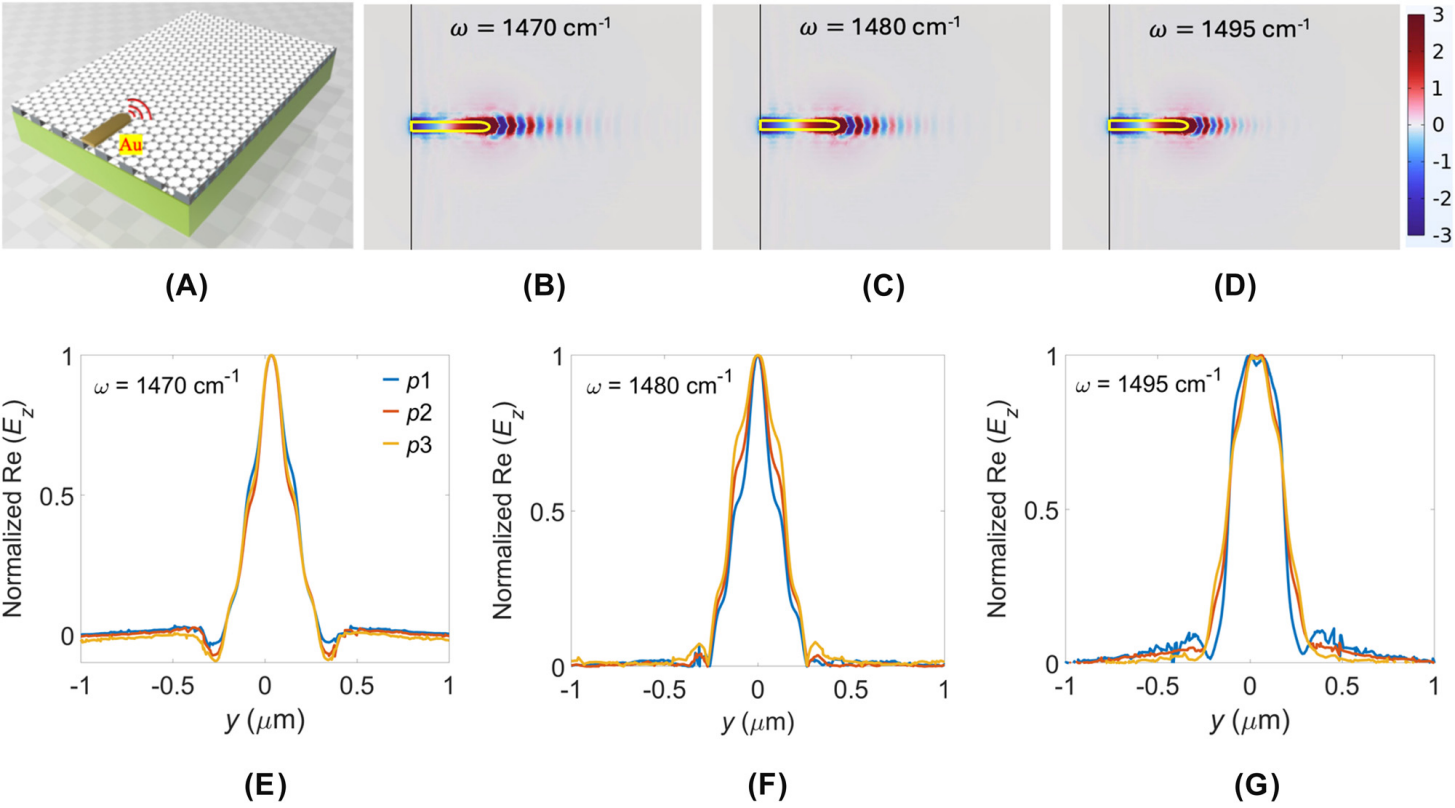
Realistic antenna-launched canalization simulation. (A) Schematic illustration of the realistic experimental setup for antenna-launched polariton canalization, employing a gold rod antenna excited by a mid-IR *s*-polarized plane wave. Simulated Re(*E*_*z*_) field distributions for frequencies *ω* = (B) 1,470, (C) 1,480, and (D) 1,495 cm^−1^ at parameters *P* = 100 nm, *w* = 70 nm, *g* = 30 nm, *h* = 20 nm, graphene Fermi energy *E*_*f*_ = 0.19 eV, and antenna tip diameter *d* = 170 nm. The computational boundaries are indicated by black vertical lines, and the antenna geometry is outlined by yellow lines. Transverse Re(*E*_*z*_) profiles at three positions for (E) *ω* = 1,470, (F) 1,480, (G) 1,495 cm^−1^ are shown to assess canalization quality.

To further evaluate the practical feasibility of our design, we simulate the excitation of HPPhPs using a realistic experimental configuration, as illustrated in Figure 5(A). Here, a gold rod antenna functions as a near-field emitter, excited by focusing a mid-IR *p*-polarized plane wave polarized parallel to the antenna’s long axis [6], [8], [19], [20], [21], [22], [24], [25], [26], [27], [28], [34], [39], [42], [43]. Practically, a tunable continuous-wave quantum cascade laser serves as the mid-IR source. Upon illumination, the antenna tip generates highly confined SPPs, which subsequently couple to PhPs in the graphene-sandwiched hBN metasurface, exciting HPPhPs. Figure 5(B)–(D) show the simulated Re(*E*_*z*_) field distributions for frequencies *ω* = 1,470, 1,480, and 1,490 cm^−1^, respectively, at parameters *P* = 100 nm, *w* = 70 nm, *g* = 30 nm, *h* = 20 nm, *E*_*f*_ = 0.19 eV, and antenna tip diameter *d* = 170 nm. In these plots, the computational boundaries are marked with black vertical lines, and the antenna geometry is outlined in yellow. The resulting field patterns agree closely with the ideal dipole source simulations presented in Figure 3(B)–(D), demonstrating consistency between practical and idealized excitation schemes.

To assess propagation characteristics, Figure S4(c) plots 1D Re(*E*_*z*_) profiles along the propagation direction (*x*-axis) starting from the antenna tip position. The calculated field decay rates exhibit minor difference compared to the ideal dipole source simulations (Figure S4(b)), confirming the robust nature of the canalization phenomenon. Additionally, Figure 5(E)–(G) compare the transverse profiles of Re(*E*_*z*_) at three positions (*p*1 to *p*3) for *ω* = 1,470, 1,480, and 1,495 cm^−1^, respectively, showing that the canalized beams excited by the antenna maintain excellent collimation. Specifically, the FWHM values at *p*3 are 250, 290, and 310 nm for *ω* = 1,470, 1,480, and 1,495 cm^−1^, respectively, confirming high-quality canalization achievable under realistic excitation conditions. These results validate the practical feasibility and tunable characteristics of the proposed graphene-sandwiched hBN metasurfaces for integrated nanophotonic and optoelectronic applications. Overall, the significances of this study include: (a) The introduction of an unprecedented mechanism to achieve orthogonal polariton canalization via plasmon–phonon polariton coupling, distinct from previously reported STO-induced canalization phenomena; (b) A canalization mode exhibiting minimal side-lobes and stable beam collimation across a wide mid-infrared spectral range (*ω* = 1,470–1,510 cm^−1^); (c) A critical evaluation demonstrating the limitations of conventional analytical approaches, such as effective medium theory (EMT) and modified EMT (MEMT), when applied to metasurfaces with strong coupling at frequencies above 1,480 cm^−1^ for the present hBN metasurfaces.

## 5 Conclusions

In summary, we have proposed and numerically demonstrated a graphene-sandwiched hBN metasurface capable of achieving high-quality and tunable canalization in the mid-infrared regime. The structure leverages STO resonances arising from strong near-field coupling among hBN ribbons, enabling the hybridization of isotropic graphene SPPs with anisotropic PhPs to form hybrid plasmon–phonon polaritons (HPPhPs). This coupling produces robust canalization propagation along a direction orthogonal to that of conventional STO-induced configurations. Simulations further reveal how the graphene Fermi energy and geometric parameters modulate canalization characteristics, maintaining narrow beam widths (∼300 nm) over a broad spectral range (1,470–1,510 cm^−1^). These findings highlight a promising pathway for tunable polariton canalization and efficient control of polariton propagation in vdW heterostructures, advancing the development of next-generation nanoscale photonic circuits.

## Supplementary Material

Supplementary Material Details
