# Supplementary material for: Orthogonal canalized polaritons via coupling graphene plasmon and phonon polaritons of hBN metasurface
Source: Nanophotonics. 2025 Nov 3;14(27):5189–98. doi: 10.1515/nanoph-2025-0385 (PMC12717929; doi:10.1515/nanoph-2025-0385)
Supplement: Supplementary file 1 — Supplementary Material Details [file j_nanoph-2025-0385_suppl_001.pdf]

Supplementary Material for "Orthogonal canalized polaritons via coupling graphene plasmon and phonon polaritons of hBN grating"

Supplementary Note 1: Permittivity of hBN and surface conductivity of graphene

The frequency-dependent permittivity $\varepsilon(\omega)$ of the hBN slab is modeled using the Lorentz oscillator model [43]:

$$\varepsilon_k(\omega) = \varepsilon_{\infty,k}\left(1 + \frac{\omega_{LO,k}^2 - \omega_{TO,k}^2}{\omega_{TO,k}^2 - \omega^2 - i\omega\gamma_k}\right), k = \perp \text{ or } \parallel \tag{S1}$$

where $\varepsilon(\omega)_\parallel$ and $\varepsilon(\omega)_\perp$ represent the out-of-plane and in-plane permittivities of hBN, respectively. The transverse optical (TO) and longitudinal optical (LO) phonon frequencies are given by $\omega_{TO,k}$ ($\omega_{TO,\parallel} = 785\ cm^{-1}, \omega_{TO,\perp} = 1395\ cm^{-1}$) and $\omega_{LO,k}$ ($\omega_{LO,\parallel} = 845\ cm^{-1}, \omega_{LO,\perp} = 1630\ cm^{-1}$) in the k direction, respectively. The damping constant is γ_k ($\gamma_\parallel = 1\ cm^{-1}, \gamma_\perp = 2\ cm^{-1}$), and the high-frequency permittivity values are $\varepsilon_{\infty,k}$ ($\varepsilon_{\infty,\parallel} = 2.8, \varepsilon_{\infty,\perp} = 3$). Based on Eqs. (1) to (3), the effective permittivity components of the hBN metasurface are plotted in Figure S1. In particular, $\varepsilon_{eq,y}$ (MEMT) is derived from the modified effective medium theory (MEMT) [38, 39] captures the strong near-field coupling between the hBN ribbons, while and $\varepsilon_{eq,y}$ (MGA) based on the Maxwell-Garnett approximation (MGA) [43, 49] reflects the case of weak coupling.

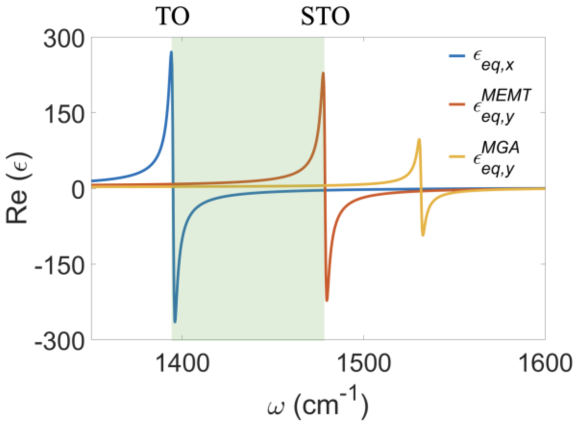

Figure S1. Effective permittivity of hBN ribbon metasurfaces. Real parts of equivalent permittivity components calculated by Eq. (1)–(3) using MEMT for a grating composed of hBN ribbons with parameters: period P = 100 nm, ribbon gap g = 30 nm, ribbon width w = 70 nm, and height h = 20 nm.

In this work, graphene is modeled as an infinitesimally thin conductive sheet, with its surface conductivity σ described by the Kubo formula [13, 32, 34, 41]:

$$\sigma = \frac{-ie^2 k_B T}{\pi\hbar^2(\omega - i/\pi)}\left(\frac{E_f}{k_B T} + 2\ln\left(e^{-E_f/k_B T} + 1\right)\right) - \frac{ie^2}{4\hbar}\ln\left(\frac{2E_f - \hbar(\omega - i/\pi)}{2E_f + \hbar(\omega - i/\pi)}\right). \quad \text{(S2)}$$

Here, ω is the angular frequency, E_f the Fermi energy, $\tau = \mu E_f / e v_f^2$ the carrier relaxation lifetime, T the absolute temperature, e the electron charge, k_B the Boltzmann constant, $\hbar$ the reduced Plank constant, μ the carrier mobility in graphene, and $v_f = 10^6$ m/s the Fermi velocity of electrons. The first and second terms in Eq. (S2) correspond to the intra-band and inter-band contributions to conductivity, respectively. The optical response of graphene can be dynamically tuned by modifying the Fermi energy E_f through electrostatic gating or chemical doping. In our simulations, we used a realistic carrier mobility of μ = 0.6 $m^2/V \cdot s$ and T = 300 K. For analytical modeling in §2, the surface conductivity σ is translated into an effective anisotropic permittivity using $\varepsilon_{gp} = 2.5 + i\,\sigma/(\omega\varepsilon_0 h_g)$ and $\varepsilon_{gz} = 2.5$, where ε_{gp} and ε_{gz} represent the in-plane and out-of-plane components of graphene's permittivity, respectively, ε_0 is the vacuum permittivity, and h_g = 0.34 nm is the nominal thickness of monolayer graphene.

Supplementary Note 2: Analytical dispersion relation of the proposed structure using a five-layer waveguide model [47]

To derive the analytical dispersion relation, the proposed graphene-covered hBN metasurface is modeled as a five-layer slab waveguide (see Fig. S2). From top to bottom, the five layers are defined as follows: air ($z > 0$), top graphene layer ($-d_1 < z < 0$), hBN ribbon grating ($-d_2 < z < -d_1$), bottom graphene layer ($-d_3 < z < -d_2$), and SiO_2 substrate ($z < -d_3$). The isotropic permittivities of the air and substrate are denoted as ε_a and ε_s, respectively. The graphene layers and the hBN metasurface are treated as anisotropic media, characterized by permittivity tensors: $\bar{\bar{\varepsilon}} = \mathrm{diag}\{\varepsilon_{gp}, \varepsilon_{gp}, \varepsilon_{gz}\}$ and $\mathrm{diag}\{\varepsilon_{eq,x}, \varepsilon_{eq,y}, \varepsilon_{eq,z}\}$, respectively. We consider polaritons propagating in the xy-plane with an in-plane wave vector $\vec{k} = (k_x, k_y)$ at an angle θ with respect to the x-axis. Under this coordinate rotation (counterclockwise about the z-axis), the effective in-plane ($\varepsilon_{\perp}$) and out-of-plane ($\varepsilon_{\parallel}$) permittivity components of the hBN metasurface are expressed as: $\varepsilon_{\perp} = \varepsilon_{eq,x}\cos^2\theta + \varepsilon_{eq,y}\sin^2\theta$ and $\varepsilon_{\parallel} = \varepsilon_{eq,z}$.

This model focuses on the derivation of the transverse magnetic (TM) mode,[34] where the magnetic field vector can be written as: $\vec{H} = H_y(z)\mathrm{e}^{(ipx-\omega t)}\hat{e}_y$ with $\hat{e}_y$ denoting the unit vector along the y-axis, and p being the propagation constant to be determined. By applying this form to the three-dimensional wave equation and considering boundary conditions across each interface, the wave equations are simplified for each layer, leading to the analytical dispersion relation of the system:

$$\frac{\partial^2 \vec{H}_y}{\partial z^2} + (k_0^2\varepsilon_{a,s} - p^2)\vec{H}_y = 0, \tag{S3}$$

$$\frac{\partial^2 \vec{H}_y}{\partial z^2} + \left(k_0^2\varepsilon_t^{(j)} - \frac{\varepsilon_t^{(j)}}{\varepsilon_z^{(j)}}p^2\right)\vec{H}_y = 0, \tag{S4}$$

where the subscript $j = 2$, 3, and 4 in Eq. (S4) refers to the layers 2 (top graphene), 3 (hBN ribbon grating), and 4 (bottom graphene), respectively. The terms $\varepsilon_t^{(j)}$ and $\varepsilon_z^{(j)}$ represent the in-plane and out-of-plane permittivity components of layer j. For convenience, we define the following relations: $\varepsilon_t^{(3)} = \varepsilon_{\perp}$, $\varepsilon_z^{(3)} = \varepsilon_{\parallel}$, $\varepsilon_t^{(2)} = \varepsilon_t^{(4)} = \varepsilon_{gp}$, and $\varepsilon_z^{(2)} = \varepsilon_z^{(4)} = \varepsilon_{gz}$. Considering the field behavior within each layer, the magnetic field component $H_y(z)$ is assumed to take the following form:

$$H_y(z) = \begin{cases} (A+B)e^{-\kappa_a z}, & (z > 0) \\ Ae^{ik_z^{(2)}z} + Be^{-ik_z^{(2)}z}, & (-d_1 < z \le 0) \\ Ce^{ik_z^{(3)}z} + De^{-ik_z^{(3)}z}, & (-d_2 < z \le -d_1) \\ Ee^{ik_z^{(4)}z} + Fe^{-ik_z^{(4)}z}, & (-d_3 < z \le -d_3) \\ (Ee^{-ik_z^{(4)}d_3} + Fe^{ik_z^{(4)}d_3})e^{\kappa_s(z+d_3)}, & (z \le -d_3) \end{cases} \tag{S5}$$

where $\kappa_{a,s} = \sqrt{p^2 - k_0^2 \varepsilon_{a,s}}$ is the transverse wave vector in air layer 1 (κ_a) or substrate layer (κ_s), and $k_z^{(j)} = \sqrt{k_0^2 \varepsilon_t^{(j)} - (\varepsilon_t^{(j)} / \varepsilon_z^{(j)}) p^2}$ is the z-component of wave vector of the layer j. The A, B, C, D, E, and F are coefficients determined by boundary conditions at the interfaces. Next, the x-component of electric field $\vec{E} = E_x(z) \mathrm{e}^{(ipx-\omega t)} \hat{e}_x$, where $\hat{e}_x$ is the unit vector in the x-direction, can be found by the curl equation $\nabla \times \vec{H} = i\omega\varepsilon_0 \tilde{\varepsilon} \vec{E}$ as follows

$$E_x(z) = \begin{cases} \frac{i\kappa_a}{\omega\varepsilon_0\varepsilon_a}(A+B)e^{-\kappa_a z}, & (z > 0) \\ \frac{k_z^{(2)}}{\omega\varepsilon_0\varepsilon_t^{(2)}}(Ae^{ik_z^{(2)}z} - Be^{-ik_z^{(2)}z}), & (-d_1 < z \le 0) \\ \frac{k_z^{(3)}}{\omega\varepsilon_0\varepsilon_t^{(3)}}(Ce^{ik_z^{(3)}z} - De^{-ik_z^{(3)}z}), & (-d_2 < z \le -d_1). \\ \frac{k_z^{(4)}}{\omega\varepsilon_0\varepsilon_t^{(4)}}(Ee^{ik_z^{(4)}z} - Fe^{-ik_z^{(4)}z}), & (-d_3 < z \le -d_3) \\ \frac{-i\kappa_s}{\omega\varepsilon_0\varepsilon_s}(Ee^{-ik_z^{(4)}d_3} + Fe^{ik_z^{(4)}d_3})e^{\kappa_s(z+d_3)}, & (z \le -d_3) \end{cases} \tag{S6}$$

To derive the mode dispersion relation, we apply the electromagnetic boundary conditions at each interface, requiring the continuity of the tangential components of the magnetic $H_y(z)$ and electric $E_x(z)$ fields. Specifically, the following conditions must be satisfied:

$$\begin{aligned} E_x^{(1)} &= E_x^{(2)}, H_y^{(1)} = H_y^{(2)}, & z_1 &= d_1 \\ E_x^{(2)} &= E_x^{(3)}, H_y^{(2)} = H_y^{(3)}, & z_2 &= -d_1 \\ E_x^{(3)} &= E_x^{(4)}, H_y^{(3)} = H_y^{(4)}, & z_3 &= -d_2 \\ E_x^{(4)} &= E_x^{(5)}, H_y^{(4)} = H_y^{(5)}, & z_4 &= -d_3. \end{aligned} \tag{S7}$$

By substituting Eqs. (S5) and (S6) into Eq. (S7), we obtain six independent equations based on the continuity conditions. Note that the conditions $H_y^{(1)}(-d_1) = H_y^{(2)}(-d_1)$ and $H_y^{(4)}(-d_3) = H_y^{(5)}(-d_3)$ are redundant due to their dependence on other boundary relations, and therefore are not included in the final set of equations.

$$\begin{bmatrix} \frac{i\kappa_a}{\varepsilon_a} - \frac{k_z^{(2)}}{\varepsilon_t^{(2)}} & \frac{i\kappa_a}{\varepsilon_a} + \frac{k_z^{(2)}}{\varepsilon_t^{(2)}} & 0 & 0 & 0 & 0 \\ \frac{k_z^{(2)}}{\varepsilon_t^{(2)}} e^{-ik_z^{(2)}d_1} & -\frac{k_z^{(2)}}{\varepsilon_t^{(2)}} e^{ik_z^{(2)}d_1} & -\frac{k_z^{(3)}}{\varepsilon_t^{(3)}} e^{-ik_z^{(3)}d_1} & \frac{k_z^{(3)}}{\varepsilon_t^{(3)}} e^{ik_z^{(3)}d_1} & 0 & 0 \\ e^{-ik_z^{(2)}d_1} & e^{ik_z^{(2)}d_1} & -e^{-ik_z^{(3)}d_1} & -e^{ik_z^{(3)}d_1} & 0 & 0 \\ 0 & 0 & \frac{k_z^{(3)}}{\varepsilon_t^{(3)}} e^{-ik_z^{(3)}d_2} & -\frac{k_z^{(3)}}{\varepsilon_t^{(3)}} e^{ik_z^{(3)}d_2} & -\frac{k_z^{(4)}}{\varepsilon_t^{(4)}} e^{-ik_z^{(4)}d_2} & \frac{k_z^{(4)}}{\varepsilon_t^{(4)}} e^{ik_z^{(4)}d_2} \\ 0 & 0 & e^{-ik_z^{(3)}d_2} & e^{ik_z^{(3)}d_2} & -e^{-ik_z^{(4)}d_2} & -e^{ik_z^{(4)}d_2} \\ 0 & 0 & 0 & 0 & (\frac{i\kappa_s}{\varepsilon_s} + \frac{k_z^{(4)}}{\varepsilon_t^{(4)}})e^{-ik_z^{(4)}d_3} & (\frac{i\kappa_s}{\varepsilon_s} - \frac{k_z^{(4)}}{\varepsilon_t^{(4)}})e^{ik_z^{(4)}d_3} \end{bmatrix} \begin{bmatrix} A \\ B \\ C \\ D \\ E \\ F \end{bmatrix} = 0. \tag{S8}$$

To obtain nontrivial solutions for the coefficients A, B, C, D, E, and F, the determinant of the coefficient matrix must be zero. This condition yields the dispersion relation of the system. By solving the resulting nonlinear equation, the propagation constant ppp can be determined as a function of the propagation angle θ.

$$
\begin{aligned}
&(m_5+m_6)\left\{\begin{matrix}(m_2+m_3)\left[m_1^2(m_3+m_4)(m_4+m_5)+(m_3-m_4)(m_4-m_5)\right]+\ldots\\(m_2-m_3)\left[m_1^2(m_3-m_4)(m_4+m_5)+(m_3+m_4)(m_4-m_5)\right]m_7^2\end{matrix}\right\}m_8^2+\\
&(m_5-m_6)\left\{\begin{matrix}(m_2+m_3)\left[m_1^2(m_3+m_4)(m_4-m_5)+(m_3-m_4)(m_4+m_5)\right]+\ldots\\(m_2-m_3)\left[m_1^2(m_3-m_4)(m_4-m_5)+(m_3+m_4)(m_4+m_5)\right]m_7^2\end{matrix}\right\}=0,
\end{aligned}
\tag{S9}
$$

where $m_1=e^{ik_z^{(3)}(d_1-d_2)}$, $m_2=\frac{i\kappa_a}{\varepsilon_a}$, $m_3=\frac{k_z^{(2)}}{\varepsilon_t^{(2)}}$, $m_4=\frac{k_z^{(3)}}{\varepsilon_t^{(3)}}$, $m_5=\frac{k_z^{(4)}}{\varepsilon_t^{(4)}}$, $m_6=\frac{i\kappa_s}{\varepsilon_s}$, $m_7=e^{ik_z^{(2)}d_1}$, $m_8=e^{ik_z^{(4)}(d_2-d_3)}$.

To obtain the analytical solution for the three-layer system corresponding to the hBN ribbon grating without graphene layers, the following nonlinear equation must be solved:

$$
(m_2+m_3)(m_3+m_6)+(m_2-m_3)(m_3-m_6)m_7^2=0. \tag{S10}
$$

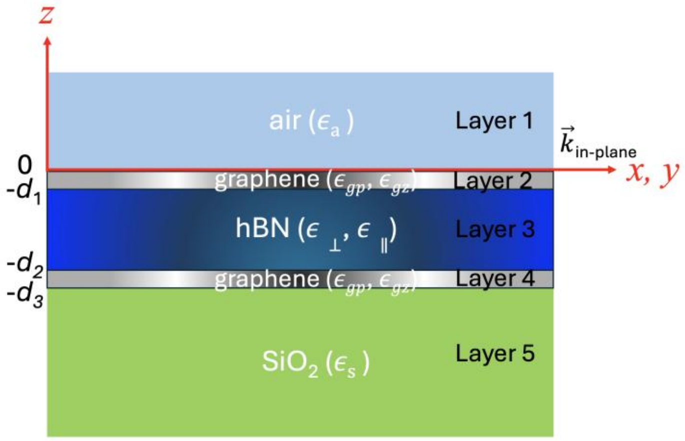

Figure S2. Five-layer waveguide model for graphene-hBN metasurfaces. Schematic of the theoretical five-layer slab waveguide model for graphene-covered hBN metasurfaces used in analytical studies. Layers 1–5 correspond respectively to air, graphene, equivalent hBN ribbon layer, graphene, and SiO_2 substrate.

Supplementary Note 3: Simulated Re(E_z) field distributions and corresponding IFCs for hBN gratings modeled with equivalent permittivity derived from the MEMT approach

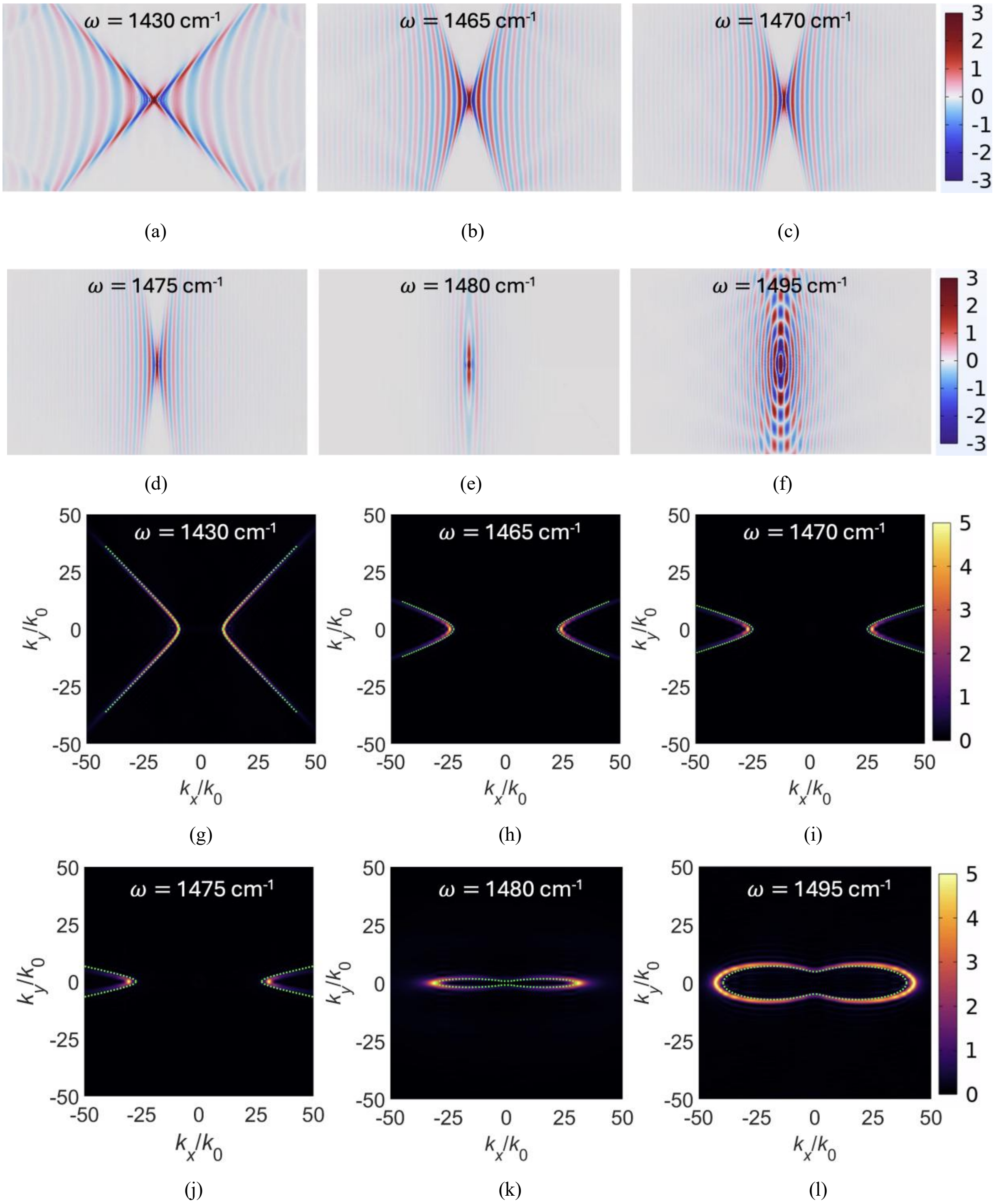

Figure S3. Numerical validation of bare hBN metasurface dispersion. Simulated Re(E_z) field distributions at frequencies ω: (a) 1430, (b) 1465, (c) 1470, (d) 1475, (e) 1480, (f) 1495 cm^{-1} for bare hBN metasurfaces. Corresponding IFCs (g)–(l) were obtained by Fourier transforming Re(E_z). Analytical solutions (green dotted lines) use the three-layer slab model [see Eq. (S10)] for comparison.

Supplementary Note 4. Simulated Re(Ez) field distributions of bare and graphene-sandwiched hBN metasurfaces along their respective canalization directions.

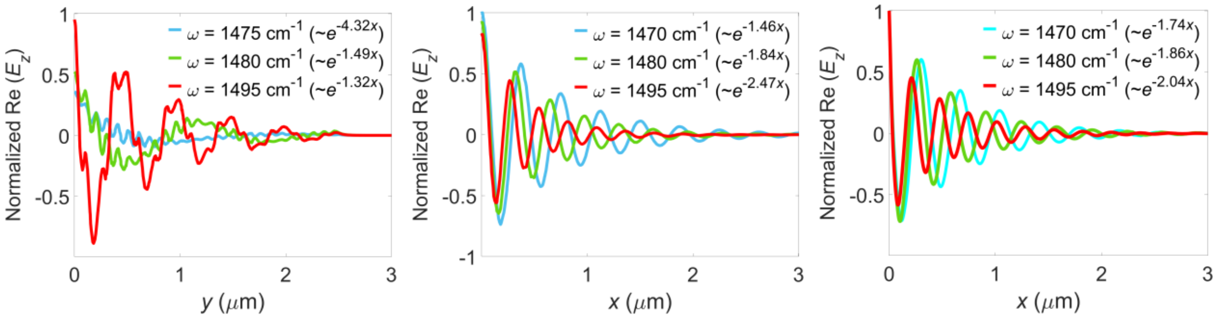

(a) (b) (c)

Figure S4. 1D profiles of Re(E_z) with the corresponding decay rates along the canalization directions. Re(E_z) field profiles along the (a) y-direction of bare hBN metasurface using an ideal dipole source at ω = 1475, 1480, and 1495 cm^{-1}, (b) x-direction of graphene-sandwiched hBN metasurface using an ideal dipole source at ω = 1470, 1480, and 1495 cm^{-1}, and (c) x-direction of graphene-sandwiched hBN metasurface using an antenna-launched source with antenna tip diameter d = 170 nm, at ω = 1470, 1480, and 1495 cm^{-1}.

Supplementary Note 5: Simulated Re(E_z) field distributions and IFCs for graphene-sandwiched hBN gratings using equivalent permittivity calculated via the MEMT

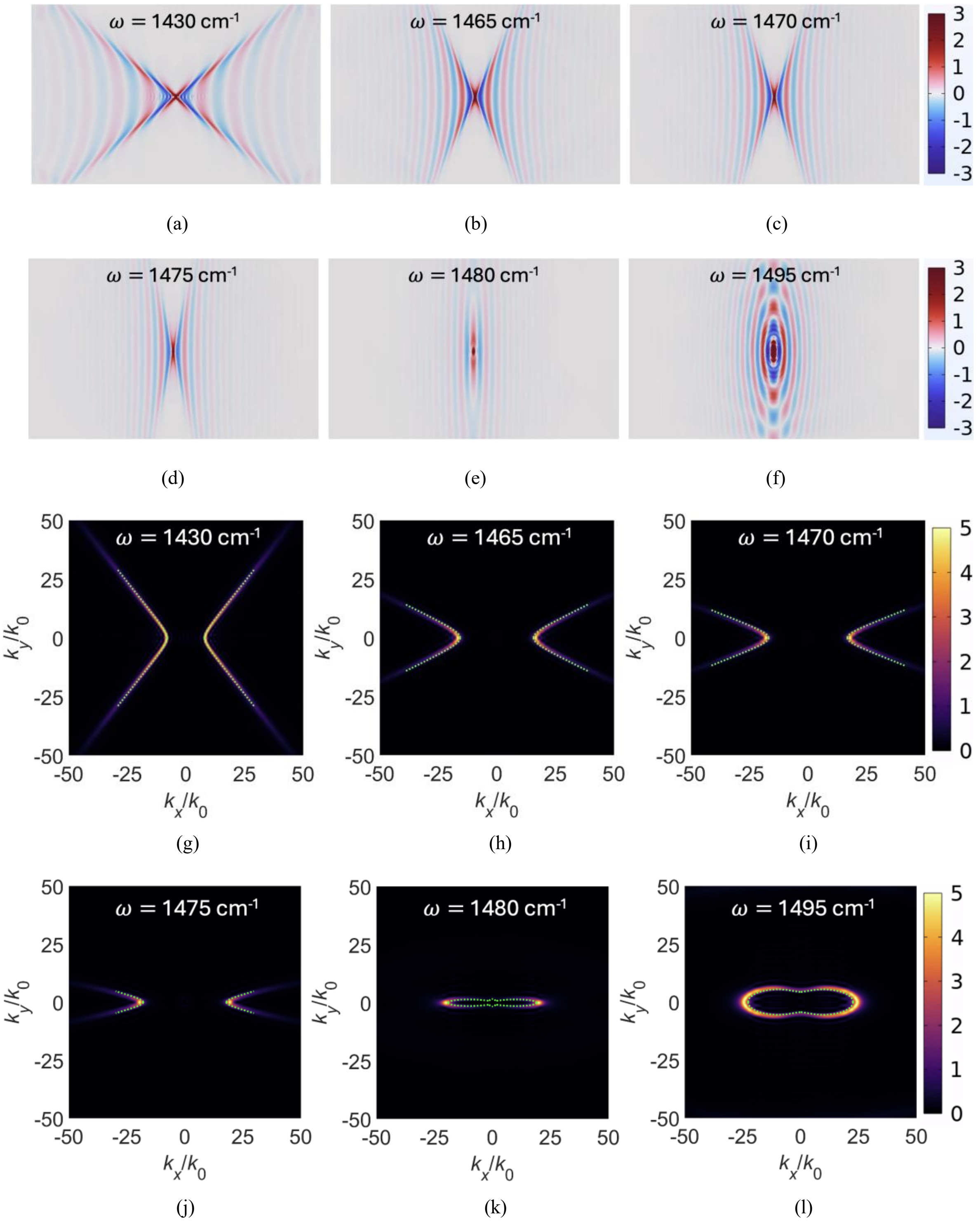

Figure S5. Numerical validation of graphene-covered hBN metasurface dispersion. Simulated Re(E_z) field distributions at frequencies ω: (a) 1430, (b) 1465, (c) 1470, (d) 1475, (e) 1480, (f) 1495 cm^{-1} for graphene-covered hBN metasurfaces, and the corresponding IFCs (g)–(l) along with analytical solutions (green dotted lines) derived from the five-layer slab model [see Eq. (S9)] under the parameters $P = 100$ nm, $w = 70$ nm, $g = 30$ nm, $h = 20$ nm, and $E_f = 0.19$ eV.

Supplementary Note 6: Simulated Re(E_z) field distributions and the corresponding IFCs of graphene-sandwiched hBN metasurface over different thickness of hBN metasurface.

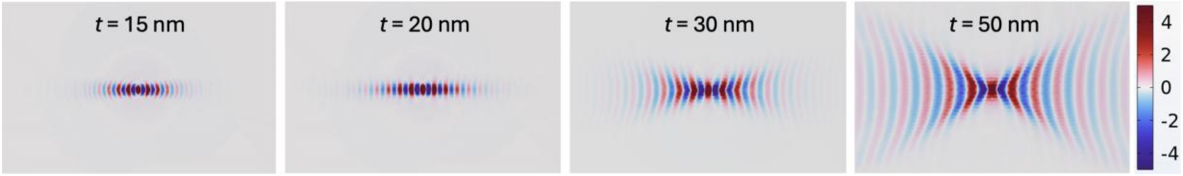

(a) (b) (c) (d)

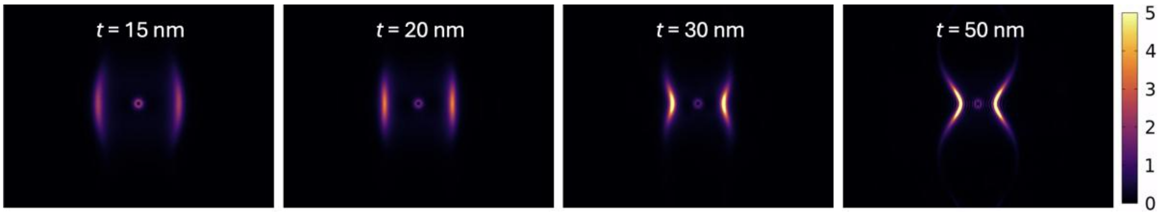

(e) (f) (g) (h)

Figure S6. Influence of hBN ribbon thickness on polariton dispersion. Thickness-dependent simulated Re(E_z) field distributions for varying hBN ribbon thicknesses t: (a) 15 nm, (b) 20 nm, (c) 30 nm, and (d) 50 nm, with their corresponding IFCs shown in (e)–(h), respectively, at the parameters ω = 1480 cm^{-1}, P = 100 nm, w = 70 nm, g = 30 nm, and E_f = 0.19 eV.
